# Povidone-iodine irrigation combined with Vancomycin powder lowers infection rates in pediatric deformity surgery

**DOI:** 10.1007/s43390-021-00333-3

**Published:** 2021-05-10

**Authors:** Rolando Figueroa Roberto, Flynn Andrew Rowan, Deepak Nallur, Blythe Durbin-Johnson, Yashar Javidan, Eric Otto Klineberg

**Affiliations:** 1 Department of Orthopedics University of California, Davis, Sacramento, USA; 2 Shriners Hospitals for Children Northern California, Sacramento, CA USA; 3 University of Indiana, Indianapolis, USA

**Keywords:** Pediatric scoliosis, Infection prevention, Topical vancomycin

## Abstract

**Background:**

Surgical site infection is a morbid, devastating complication after spinal procedures. Studies have investigated the effect of wound lavage with 3.5% Povidone-iodine solution or the use of intrawound Vancomycin powder. We examined the effect of Povidone-iodine irrigation, intrawound Vancomycin powder, or a combination of both agents in a tertiary care Pediatric Hospital.

**Methods:**

We queried our health system database for patients undergoing spinal surgery over an eight-year span between January 2008 and June 2016 and identified patient cohorts who received no intervention, intrawound Vancomycin alone, Povidone-iodine irrigation alone, or a combination of both agents. Infection rates were determined. The effect of treatment on outcome was analyzed using a logistic regression model.

**Results:**

475 patients were identified who met study inclusion criteria. 88 non-neuromuscular patients received no intra-operative agent. The surgical site infection (SSI) rate in this group of patients was 10%. For the 194 non-neuromuscular scoliosis patients who received Povidone-iodine and Vancomycin powder, the infection rate was reduced to 0.7%. The SSI rate in the 180 non-neuromuscular patients who were treated with Vancomycin powder alone was 1.4%. 13 patients were treated with Povidone-iodine lavage only, with a small sample size precluding statistical comparison. Infection rate in the 132 neuromuscular disease patients decreased from 14 to 7% overall during this time span: while the odds ratio of infection was reduced in all neuromuscular treatment groups receiving intra-operative measures, statistical significance was not reached in any neuromuscular group studied.

**Conclusions:**

A protocol using combined 3.5% weight/volume Povidone-iodine and Vancomycin powder was associated with the lowest infection rate in our non-neuromuscular patient population and should be considered as a low cost intervention in pediatric patients undergoing spinal deformity procedures.

**Level of evidence:**

Level II.

## Introduction

Surgical site infection developing after operation has been a major source of patient harm, afflicting patients of all age groups and across all surgical disciplines. While the risk of SSI may have been underestimated by individual practitioners and health systems, the magnitude of this problem has not been hidden from national scrutiny. For example, a Centers for Disease Control and Prevention survey conducted in 2015 revealed that in 2015 alone 110,800 surgical site infections occurred after inpatient surgical procedures. SSI following spinal surgery is a serious complication associated with poor outcomes including prolonged hospitalization, hospital readmission, re-operation, need for long-term antibiotics, decreased quality of life, and increased cost of care in adult populations [1–7]. While there are fewer studies examining pediatric spinal deformity patients, SSI rates are lowest among those with Adolescent Idiopathic Scoliosis (AIS) ranging from 0.5–6.7%. In contrast, SSI rates as high as 42% have been reported in patients with an underlying neuromuscular condition, such as Cerebral Palsy, Muscular Dystrophy, or Spina Bifida [8].

Numerous antimicrobial prophylaxis protocols have been implemented and studied whose aim is to reduce the rate of wound infection in adult spinal surgery populations. For example, positive effects have been reported in operative series using intra-operative wound irrigation with dilute Povidone-iodine solution. Additionally, there has been much interest and research published examining the effects of topical application of Vancomycin powder prior to wound closure in spinal deformity patients. In a recent study of 1732 patients undergoing instrumented thoracolumbar fusions, the use of intrawound Vancomycin reduced surgical site infection rates from 2.6 to 0.2% [9]. Other studies corroborate the associated reduction of SSI when Vancomycin powder is applied intra-operatively in spinal surgery patients [10–12]. Wound lavage with antimicrobial solutions has also demonstrated positive effects in the reduction of postoperative surgical site infection. For example, wound lavage with a dilute Povidone-iodine solution has been shown to reduce rates of surgical site infection. In a study of 414 adult patients, the use of a 3.5% Povidone-iodine irrigation led to a decrease in the rate of SSI from 3.4 to 0% [13].

Our group previously published on the use of both intrawound Vancomycin powder and dilute Povidone-iodine irrigation in an adult population, demonstrating a 50% reduction in surgical site infection [1].

In that study, we found a significant reduction in both the rate of methicillin-resistant Staphylococcus aureus (MRSA) and polymicrobial infections. Given our success in reducing SSI in our adult spinal surgery patient population, we instituted a similar protocol with dual antimicrobial intra-operative prophylaxis at our associated pediatric hospital in 2013. The purpose of this study was to assess the efficacy of Povidone-iodine irrigation combined with intrawound Vancomycin powder in reducing SSI in pediatric patients undergoing spinal deformity surgery.

## Materials and methods

A retrospective review was conducted of all patients who underwent spinal fusion between January 2008 and June 2016. We compared patients treated prior to the introduction of an intraoperative prophylaxis program (no intraoperative prophylaxis), patients treated during the adoption of the protocol, and patients treated after the universal adoption of a protocol combining wound lavage with Povidone-Iodine and placement of topical Vancomycin powder prior to wound closure. Specifically, we used a minimum of 3 L of Betadine solution consisting of 30 cc of Povidone Iodine per liter of irrigation administered with a pulsatile lavage device. With regards to Vancomycin powder application, Adolescent patients of normal stature were treated by sprinkling 2 g of antibiotic powder in the deep muscle and implant layer after decortication and bone grafting and prior to wound closure. Small statured patients and small wounds, eg. Hemivertebra excision in a 4-year-old patient would be treated with 1 g of Vancomycin powder in the deep tissue layer prior to wound closure. No attempt was made to place antibiotic powder in the subcutaneous interval. During the 12 month period of protocol initiation, there was incomplete adherence to the dual antimicrobial prophylaxis regimen; hence, patients may have been treated with only one of the therapeutic agents. This resulted in four patient treatment groups—those treated with no intrawound antimicrobial agent, those treated with only 3.5% weight/volume Povidone-iodine irrigation, those treated with only intrawound Vancomycin powder, and those treated with both Povidone-iodine irrigation and intrawound Vancomycin powder. All pediatric age group patients undergoing spinal deformity surgery at a single institution were identified through hospital records; diagnostic codes for scoliosis etiology were verified to allow subgroup analysis by scoliosis type. Patients were excluded if they had insufficient records, i.e. less than 6 months of follow-up, or surgery that did not involve a multilevel instrumented fusion (e.g. micro-discectomy or single-level fusion for spondylolisthesis). Patient records including preoperative history and physical, operative reports, intra-operative nursing reports, postoperative records, and microbiological laboratory reports were used for data collection. Use of Vancomycin powder or dilute Povidone-iodine irrigation was confirmed by documentation in either the operative report or nursing record. During the period of study all patients were screened for MRSA nares infections as per State Department of Health mandates, however, other than isolation precautions there was no attempt to eradicate topical colonization. The effect of treatment on the probability of infection was analyzed using a logistic regression model with effects for treatment, scoliosis type (neuromuscular or other), and the interaction between treatment and scoliosis type. Model parameters were estimated using Firth’s bias-reduced logistic regression [14], due to the small numbers of infections in some patient categories.

Analyses were conducted using the statistical software environment R, version 3.4.1, [15] and Firth’s bias-reduced logistic regression was conducted using the R package, version 1.2.2.

## Results

In total there were 475 patients who met study inclusion criteria whose data was reviewed and included in the analysis. Table 1 summarizes patient characteristics by treatment group. Of the 475 patients, eighty-eight did not receive antibiotic powder or Betadine lavage, thirteen patients underwent irrigation with 3.5% Povidone-iodine irrigation solution only, one hundred and eighty patients received intrawound Vancomycin powder only, and one hundred and ninety-four patients were treated with both a dilute Povidone-iodine wound lavage protocol and intrawound Vancomycin powder.

**Table 1:** Patient characteristics by treatment group

	Neither (*n* = 88)	Povidone-iodine only (*n* = 13)	Vancomycin only (*n* = 180)	Both (*n* = 194)	All patients (*n* = 475)
Age at surgery					
*N*	88	13	180	194	475
Mean (SD)	13.3 (4.5)	13.1 (3.4)	13.9 (3.5)	13.9 (3.2)	13.8 (3.6)
Median (range)	14.1 (1.6–21)	13.5 (7.4–17.4)	14.4 (2.7–19.9)	14.2 (2.1–20.7)	14.2 (1.6–21)
Gender (*n*, %)					
F	48 (54.5%)	8 (61.5%)	126 (70%)	128 (66%)	310 (65.3%)
M	40 (45.5%)	5 (38.5%)	54 (30%)	66 (34%)	165 (34.7%)
Scoliosis type (*n*, %)					
Congenital	19 (21.6%)	3 (23.1%)	30 (16.7%)	21 (10.8%)	73 (15.4%)
Idiopathic	27 (30.7%)	5 (38.5%)	96 (53.3%)	101 (52.1%)	229 (48.2%)
Juvenile scoliosis	2 (2.3%)	1 (7.7%)	3 (1.7%)	5 (2.6%)	11 (2.3%)
Neuromuscular	35 (39.8%)	3 (23.1%)	40 (22.2%)	54 (27.8%)	132 (27.8%)
Scheuermann's	5 (5.7%)	1 (7.7%)	11 (6.1%)	13 (6.7%)	30 (6.3%)

The average patient age was thirteen with a slight female predominance in all treatment groups. Idiopathic scoliosis was the most common type of scoliosis in all treatment groups (range 30–53%), with neuromuscular scoliosis accounting for approximately one-third of all patients in each treatment group (23–39%), and congenital scoliosis accounting for 10–20% of patients studied. Juvenile idiopathic scoliosis and Scheuermann's disease patients represented approximately 2% and 6% of patients respectively in the treatment cohorts.

Of the 88 patients who received no intra-operative measures there were nine patients with postoperative deep surgical site infection (SSI), demonstrating a 10% overall infection rate. In the no prophylaxis group, five of 35 neuromuscular patients became infected (14% infection rate) while only four of 53 non-neuromuscular patients were diagnosed with deep SSI (7.5% infection rate). In the smallest sample group, none of the 13 patients who received only wound lavage with a dilute Povidone-iodine solution became infected. Three of 40 neuromuscular patients who received intra-wound Vancomycin powder only were diagnosed with deep SSI (7.5% infection rate) and two of 140 patients with non-neuromuscular diagnoses (eg. Congenital, idiopathic, Scheuermann’s) treated with Vancomycin powder alone developed deep SSI (1.4%). One of 140 patients (0.7%) in the non-neuromuscular group treated with both Povidone-iodine lavage and intrawound Vancomycin powder developed a deep SSI. Infection developed after an operation in four of 54 (7.4%) neuromuscular scoliosis patients despite treatment with a combined protocol of lavage and intrawound Vancomycin powder.

Table 2 shows infection rates by treatment group and scoliosis type dividing the scoliosis types into neuromuscular etiology and non-neuromuscular etiology. We combined the more uncommon types of spinal deformity diagnoses including Congenital scoliosis, juvenile idiopathic scoliosis and Scheuermann’s kyphosis with adolescent idiopathic scoliosis into one non-neuromuscular group as the statistical analysis of groups into sizes ranging between 5 and 20 precluded individual statistical analysis (see Tables 2 and 3 for groupings and subgroups). Our neuromuscular group on the other hand included patients with Cerebral Palsy, Spinal cord injury, any one of several muscular dystrophies, myopathies, myelomeningocoele, and other rare neurological disorders of childhood. Without exception all of these patients underwent posterior spinal fusions from T2 to the pelvis and were non ambulators. Table 3 demonstrates the infection rates and treatment strategies when parsing the non-neuromuscular groups into the smallest individual diagnostic categories. The resultant division results in very small treatment groups precluding reasonable statistical analysis of the smallest groups.

**Table 2:** Infection rates by treatment and scoliosis type: number of infections in each group (% infections rate) vs. intra-operative prophylactic measure

Dichotomous grouping of neuromuscular vs. non-neuromuscular		
	Neuromuscular	Non neuromuscular (congenital, idiopathic, scheuermann’s)
Neither (*n* = 88)	5/35 (14.3%)	4/53 (7.5%)
Povidone-iodine only (*n* = 13)	0/3	0/10
Vancomycin only (*n* = 180)	3/40 (7.5%)	2/140 (1.4%)
Both (*n* = 194)	4/54 (7.4%)	1/140 (0.7%)

**Table 3:** Infection rates by treatment and scoliosis type

	Neuromuscular	Congenital	Adolescent idiopathic	Juvenile	Scheurmann’s
Neither (*n* = 88)	5/35 (14.3%)	1/19 (5.3%)	2/27 (7.4%)	0/2	1/5 (20%)
Povidone-iodine only (*n* = 13)	0/3	0/3	0/5	0/1	0/1
Vancomycin only (*n* = 180)	3/40 (7.5%)	1/30 (3.3%)	0/96	1/3 (33.3%)	0/11
Both (*n* = 194)	4/54 (7.4%)	0/21	1/101 (1.0%)	0/5	0/13

Infection rates were highest in patients with a neuromuscular disease with the highest SSI rate seen in neuromuscular patients who received no prophylaxis (14.3% infection rate). The lowest SSI rate was seen in patients with non-neuromuscular diagnoses who were treated with a combination of dilute Povidone Iodine lavage and Vancomycin powder; in contrast, non-neuromuscular patients receiving no prophylaxis experienced a 7.5% SSI rate. The logistic regression model (Table 4) demonstrates that the difference in infection rate between no intra-operative and dual intra-operative prophylaxis *is* statically significant. We were unable to demonstrate statistically significant effects of our interventions in the neuromuscular groups due to small sample size.

**Table 4:** Logistic Regression analysis of probability of infection by treatment results for all patients

	Odds ratio	95% confidence inter-val for odds ratio	*p* value
Povidone-iodine only vs. neither	0.64	(0.06, 6.85)	0.715
Vancomycin only vs. neither	0.32	(0.11, 0.94)	0.038
Both vs. neither	0.24	(0.08, 0.77)	0.016
Vancomycin only vs. Povidone-iodine only	0.50	(0.04, 5.58)	0.571
Both vs. Povidone-iodine only	0.38	(0.03, 4.37)	0.434
Both vs. Vancomycin Only	0.75	(0.21, 2.66)	0.661

Table 4 shows estimates from the logistic regression model of the main effect of treatment on the probability of infection when all pediatric scoliosis types were combined (*n* = 475). The positive effects of the treatment effect of a dual protocol (Povidone-iodine and Vancomycin) and single-agent intra-wound Vancomycin powder compared to no intra-operative intervention is best demonstrated in this logistic regression model. The Odds ratio of postoperative wound infection in the dual intra-operative therapy group compared to the no prophylaxis group is 0.24 (95% CI 0.08–0.77, *p* = 0.016). Similarly, statistically significant results were demonstrated when comparing Vancomycin powder administration compared to no agent on the likelihood of surgical site infection Odds Ratio 0.32 (95% CI 0.11–0.94, *p* = 0.038). The remaining comparisons did not demonstrate significant treatment effects on the likelihood of infection and are summarized in Table 4.

### Treatment effects neuromuscular patients only

Table 5 shows estimates from the logistic regression model of the effect of treatment on the probability of infection in neuromuscular patients. Treatment was not significantly associated with a reduction in the infection rates for these patients. While antibacterial wound lavage and Vancomycin powder in combination were associated with a lower overall infection rate 7% rate vs. 14% when no prophylaxis was used, we could not demonstrate statistical significance when analyzed.

**Table 5:** Logistic regression analysis of probability of infection by treatment results for neuromuscular patients (*n* = 132)

	Odds ratio	95% confidence inter-val for odds ratio	*p* value
Povidone-iodine only vs. neither	0.79	(0.02, 27.4)	0.897
Vancomycin only vs. neither	0.52	(0.12, 02.19)	0.371
Both vs. neither	0.49	(0.13, 1.89)	0.302
Vancomycin only vs. Povidone-iodine only	0.65	(0.02, 23.8)	0.817
Both vs. Povidone-iodine only	0.62	(0.02, 21.9)	0.795
Both vs. Vancomycin only	0.95	(0.22, 4.18)	0.951

### Treatment effects non-neuromuscular patients (idiopathic scoliosis, Scheuermann's kyphosis and congenital scoliosis)

As shown in Table 6, differences were noted in non-neuromuscular patients who received intra-operative prophylactic measures. The lowest SSI rate was seen in patients who received both intra-wound Vancomycin and Betadine lavage who were compared to those receiving no prophylaxis (Odds ratio 0.12, 95% CI 0.02–0.78, *p* = 0.027). In non-neuromuscular patients who received Vancomycin powder alone there was a marked reduction in SSI rate compared to those who received no intra-operative prophylaxis. (Odds Ratio 0.20 95% CI 0.04–0.97, *p* = 0.046).

**Table 6:** Logistic regression analysis of probability of infection by treatment results for non-neuromuscular patients (*n* = 343)

	Odds ratio	95% confidence inter-val for odds ratio	*p* value
Povidone-iodine only vs. neither	0.52	(0.02, 12.0)	0.686
Vancomycin only vs. neither	0.20	(0.04, 0.97)	0.046
Both vs. neither	0.12	(0.02, 0.78)	0.027
Vancomycin only vs. Povidone-iodine only	0.38	(0.02, 9.58)	0.556
Both vs. Povidone-iodine only	0.23	(0.01, 6.67)	0.389
Both vs. Vancomycin only	0.60	(0.08, 4.60)	0.62

## Discussion

Surgical site infection causes patient harm and jeopardizes patient outcomes in all age groups and across all surgical disciplines. The magnitude of this problem has recently been the source of extensive focus by administrative bodies and insurance programs. For example, a Centers for Disease Control and Prevention survey conducted revealed that in 2015 alone 110,800 surgical site infections occurred after inpatient surgical procedures. At the outset of this intervention, we were faced with an unacceptably high surgical site infection rate and an opportunity to make a significant clinical difference. Statistical analysis in our investigation was aided by a baseline SSI rate which ranged between 7 and 14%; i.e., our ability to adequately power this study provided opportunities that did not exist at centers that had already achieved exemplary infection rates.

While the use of Vancomycin powder had been documented to decrease SSI in adult populations, and Povidone-iodine irrigation had been shown to decrease SSI in adult populations, to our knowledge, this is the first study to assess the use of both interventions using a standardized protocol for reducing surgical site infection in a pediatric population. Our results and conclusions differ substantially from a recent report from another Pediatric Tertiary Care referral center [16]. In this retrospective Pediatric series, patients with cervical disease, tumor, trauma and single-level surgeries for spondylolysis or spondylolisthesis are included. These authors specifically did not observe a positive effect on Vancomycin powder with regards to SSI reduction, however, infection rates were only able to be reduced to 2–3% which remains higher than in our non -neuromuscular population.

Other centers have similarly reported on intra-operative measures to reduce infection using similar methods to our own. A smaller series of 116 scoliosis patients was divided into Gentamicin irrigation, Betadine irrigation and a third treatment limb with instillation of Vancomycin powder after Betadine irrigation [17]. Treatment groups were small with only 32 patients in the combined therapy group. The overall infection rate in the Gentamycin irrigation group was 26%, in the Betadine wash group 7% and 6.3% in the Betadine plus Vancomycin powder group. These authors ultimately concluded that there was no benefit to the addition of Vancomycin powder. It should be noted that their infection rates are still unacceptably high on a a path towards a 0% infection rate and one wonders whether their study was adequately powered given the small number of patients in each treatment limb.

The results of our study are at this point unique in supporting the hypothesis that using intra-wound Vancomycin powder after Povidone-iodine irrigation is effective in reducing SSI in pediatric spinal deformity surgery. Our combined protocol when analyzed in aggregate demonstrated clinically significant reduction in infection rates, (7.5–0.7%) with robust statistical significance. Although the overall infection rate in neuromuscular patients decreased by approximately one half once dual intra-operative measures were initiated (14.3–7.4%) this result was not statistically significant and we can make no conclusions as to cause and effect in this patient group. Given the much smaller sample size in our study, our failure to reach significance may be related to statistical power. However, we also propose that an antibiotic agent with more gram-negative coverage would be necessary to prevent SSI in this group of patients who more commonly suffer from chronic urinary colonization.Further, sterilization of the deep wound environment may not be sufficient to prevent infection in the neuromuscular deformity patient population. For example, in the Spina Bifida population, attenuated soft tissue envelopes from myelo sac closure may overwhelm the treatment effect of antimicrobial interventions. In these patients, wound dehiscence results in contamination with urinary and fecal flora given the proximity of a complex spinal wound with the perineum and rectum.

We cannot rule out the possibility that treatment effects are not responsible for the direct reduction in infection rates (Type 1 error). This study also suffers from a substantial sample size imbalance due to the small number of patients treated with Povidone-iodine lavage alone. A truly randomized control trial with equal treatment groups might result in greater statistical significance in more of the treatment groups. In general, aggressive preventive measures are advocated for all patients at risk for SSI, hence, multimodal treatment has been adopted in response to local, statewide and federal insurance plans who have mandated reduced complication rates.

### Other study limitations

Our study does have all of the shortcomings associated with a retrospective study. For example, while we aim to optimize patient risk factors for infection including visceral protein stores, body mass index and weight, we do not have a prospective cohort matched for these parameters. While we would ideally match patients for curve size, Hemoglobin, hematocrit, albumin level, and treatment modalities in a prospective randomized study, we have felt pressured by external agencies to push toward perfection in the avoidance of SSI, that is to achieve a net-zero SSI occurrence rate. As in any clinical practice, we frequently are driven to operate on worsening scoliosis in patients in whom determinants of health (eg. poverty, affluence, poor health habits) are varied and cannot be controlled. As such we have reported on an “all patients” cohort as we feel compelled to understand how we are doing as an entire practice and health care entity. As clinicians at a free-standing hospital, it is imperative that we can inform our patients of the precise risk of infection for all patients undergoing operation at our institution.

## Conclusions

Surgical Site Infection rate in a pediatric deformity population was reduced most effectively when we combined the use of 3.5% weight/volume Povidone-iodine lavage with intra-wound Vancomycin powder. The treatment effect was significantly demonstrated in non-neuromuscular patient (Odds ratio 0.12 when compared to no prophylaxis). Povidone-iodine alone and Vancomycin alone were independently associated with lower SSI rates in non-neuromuscular disease patients (Odds ratio 0.52 and 0.20 respectively). Our protocol was not successful in neuromuscular disease patients who included cerebral palsy, spina bifida, various muscular dystrophies or spinal cord injury associated scoliosis. While the infection rate declined from 14.3–7.4% in neuromuscular disease patients during the time period of study, we are furthest from a “never event” in this group of at risk patients. Follow-up studies should be performed to reduce the wound complication/infection rate in this most frail pediatric deformity subpopulation.
